# Automated DNA Sequence-Based Early Warning System for the Detection of Methicillin-Resistant *Staphylococcus aureus* Outbreaks

**DOI:** 10.1371/journal.pmed.0030033

**Published:** 2006-01-10

**Authors:** Alexander Mellmann, Alexander W Friedrich, Nicole Rosenkötter, Jörg Rothgänger, Helge Karch, Ralf Reintjes, Dag Harmsen

**Affiliations:** 1Institute for Hygiene, University Hospital Münster, Münster, Germany; **2**Hamburg University of Applied Sciences, Hamburg, Germany; **3**Ridom GmbH, Würzburg, Germany; **4**Department of Periodontology, University Hospital Münster, Münster, Germany; University College LondonUnited Kingdom

## Abstract

**Background:**

The detection of methicillin-resistant *Staphylococcus aureus* (MRSA) usually requires the implementation of often rigorous infection-control measures. Prompt identification of an MRSA epidemic is crucial for the control of an outbreak. In this study we evaluated various early warning algorithms for the detection of an MRSA cluster.

**Methods and Findings:**

Between 1998 and 2003, 557 non-replicate MRSA strains were collected from staff and patients admitted to a German tertiary-care university hospital. The repeat region of the *S. aureus* protein A *(spa)* gene in each of these strains was sequenced. Using epidemiological and typing information for the period 1998–2002 as reference data, clusters in 2003 were determined by temporal-scan test statistics. Various early warning algorithms (frequency, clonal, and infection control professionals [ICP] alerts) were tested in a prospective analysis for the year 2003. In addition, a newly implemented automated clonal alert system of the Ridom StaphType software was evaluated.

A total of 549 of 557 MRSA were typeable using *spa* sequencing. When analyzed using scan test statistics, 42 out of 175 MRSA in 2003 formed 13 significant clusters (*p* < 0.05). These clusters were used as the “gold standard” to evaluate the various algorithms. Clonal alerts (*spa* typing and epidemiological data) were 100% sensitive and 95.2% specific. Frequency (epidemiological data only) and ICP alerts were 100% and 62.1% sensitive and 47.2% and 97.3% specific, respectively. The difference in specificity between clonal and ICP alerts was not significant. Both methods exhibited a positive predictive value above 80%.

**Conclusions:**

Rapid MRSA outbreak detection, based on epidemiological and *spa* typing data, is a suitable alternative for classical approaches and can assist in the identification of potential sources of infection.

## Abstract 2

Harmsen and colleagues show that an early warning algorithm based on epidemiological and DNA typing is a feasible approach for detection of clusters of MRSA infection.

## Introduction

In the United States alone, infections acquired in hospitals affect 2 million patients, account for half of all major hospital complications, and result in annual costs of more than $4.5 billion [1]. *Staphylococcus aureus* is the leading cause of these nosocomial infections that include a wide range of diseases such as endocarditis, septicemia, skin infections, soft tissue infections, and bone infections [2]. Strains resistant to methicillin, in particular, have become a major concern in the hospital environment because of the high mortality rate and the stringent hygienic requirements needed for patients who are harboring a methicillin-resistant *S. aureus* (MRSA) [3,4]. Moreover, since the emergence of strains that are insensitive or have reduced sensitivity to glycopeptides, there is a real danger of infections spreading that have even greater drug resistance [5].

Analysis of laboratory test results and patients' charts are the methods usually used to identify outbreaks. However, the manual review of laboratory test results is time-consuming and resource-intensive. Electronic analysis of data can help identify suspicious patterns of disease and antimicrobial resistance [6], but such sentinel methods are rarely used in clinical practice. The typing of MRSA isolates, not only from clinical specimens, but also from surveillance cultures, is necessary for the elucidation of possible transmission routes. Because the procedures are slow and laborious, molecular typing (e.g., pulsed-field gel electrophoresis [PFGE]) is usually used a posteriori to track the course of nosocomial infections in an already established outbreak. Furthermore, PFGE requires great efforts to harmonize protocols and is therefore only partially successful in generating reproducible results [7]. In order to improve the speed of typing, DNA sequence-based approaches, such as the multi-locus sequence typing (MLST), are becoming more frequently used [8]. However, MLST is not suitable for routine surveillance of MRSA because of the high costs involved and the low discriminatory power compared to PFGE. Frenay et al., who were the first to use a single-locus sequence typing method for *S. aureus,* employed the sequence of the polymorphic region X of the *S. aureus* protein A gene *(spa)* for typing [9]. Since then, numerous studies evaluated this variable number of tandem repeat targets as quite suitable for short-term epidemiological applications, e.g., [10–13]. Because of the paucity in software for repeat identification and lack of a consensus in assigning *spa* type names, the wide-spread use of the method was hampered for years until the recent introduction of the Ridom StaphType software [14]. With this software, the *spa* sequences are analyzed automatically and linked to a database integrated with epidemiological information. A universal nomenclature is achieved by synchronization with a central server that assigns new *spa* types for all users (http://www.spaserver.ridom.de).

The aim of the study reported here was therefore to analyze the utility of a *spa* sequence-based, automatic early warning algorithm to detect MRSA clusters in hospitals and to compare this approach with classical surveillance techniques. We hypothesized that the automated system, once established, can complement and even replace the labor-intensive traditional methods used for cluster identification.

## Methods

### Setting

Between 1998 and 2003, a total of 557 non-replicate MRSA isolates were collected at the University Hospital Münster (UHM), Germany, a 1,480-bed tertiary-care teaching facility. In 2003, there were approximately 43,000 annual admissions to the hospital where the mean length of stay was 9.8 d. The prevalence of patients with MRSA colonizations and infections was taken as the annual number of persons harboring MRSA (×100) divided by the total number of admissions at UHM [15]. The baseline for calculation of the relative risk was the year 1998.

### Surveillance and Infection Control Measures

All new MRSA cases were monitored prospectively by infection control professionals (ICP) from the day when MRSA was first identified until hospital discharge. Information on each patient was obtained by reviewing medical records and laboratory data and holding telephone interviews with the attending physician. Subsequently, the ICP decided if a transmission event was likely and if further investigation was necessary. In more detail, the following infection control measures were implemented: (i) As recommended in the guidelines of the Robert Koch Institute (Berlin, Germany), all patients infected or colonized with MRSA were placed in contact isolation until the time of discharge or until eradication could be documented in three consecutive sets of negative surveillance cultures (separated by at least 24 h). MRSA surveillance cultures included swabs of several body sites (nose, groin, skin lesions, inguinal, perineal, and axillary swabs). In the case of infected patients, samples were taken from the site of infection. (ii) All patients known to have been previously colonized or infected with MRSA were isolated on re-admission to UHM and surveillance swabs were obtained. Negative surveillance cultures were mandatory in order to terminate contact isolation. (iii) Clinical microbiology laboratory results were monitored daily for the occurrence of specimens containing MRSA. (iv) *spa* typing of MRSA isolates, as performed since 2002, were carried out directly after detection of a new MRSA isolate. (v) Colonized patients were treated with nasal mupirocin ointment for 5 d and daily chlorhexidine body washes were applied. In the case of patients remaining in the hospital after eradication, weekly surveillance cultures were recommended over a 4-wk period, and then at monthly intervals to detect possible re-colonization. (vi) To detect MRSA colonization and cross-transmission, surveillance cultures were obtained from roommates as soon as a new MRSA patient was identified. (vii) Staff were screened when nosocomial transmissions were suspected and at intervals as a surveillance method on high-risk wards. (viii) Hospital staff found MRSA-positive were suspended from work on the wards until the successful eradication of MRSA could be documented. (ix) Systematic surveillance cultures at the time of admission and on a weekly basis thereafter were begun in 2002 in wards caring for high-risk patients, e.g., intensive care units [15,16]. Colonization and infection were defined in accordance with the Centers for Disease Control and Prevention criteria [17].

### Microbiology and Molecular Typing

The strain collection consisted of MRSA from various clinical sources (e.g., blood cultures and wound infections) and included surveillance cultures from patients and staff. Of all clinical *S. aureus* isolates, 6.4% exhibited methicillin resistance in 2003. For species identification, every strain was tested with API ID 32 Staph (bioMérieux, Marci l'Etoile, France) and for the presence of free coagulase. The presence of the *mecA* gene responsible for methicillin resistance was confirmed using PCR [18]. The sequence of the short sequence repeat region of the *spa* gene encoding the *S. aureus* protein A was determined in 557 strains [14]. The primers spa-1113f (5′- TAA AGA CGA TCC TTC GGT GAG C −3′) and spa-1514r (5′- CAG CAG TAG TGC CGT TTG CT −3′) were used for *spa* amplification and Taq Cycle sequencing. DNA sequences were obtained with an ABI Prism 3100 Avant Genetic Analyzer (Applied Biosystems, Foster City, California, United States) and analyzed with the Ridom StaphType software version 1.5 beta (Ridom GmbH, Würzburg, Germany) incorporating the newly added automated early warning system (“clonal alerts”) for MRSA cluster detection [14]. Typability, discriminatory index, and the 95% confidence interval (CI) of the discriminatory index were calculated using the procedures published previously [19,20].

### Retrospective Temporal-Scan Test Statistics

To evaluate the various early warning algorithms, we performed scan test statistics using the epidemiological and typing information from 1998 to 2002 as historical data to determine MRSA clusters in 2003 [21,22]. Temporal-scan statistics evaluates whether an apparent cluster of disease is unlikely to occur by chance alone. Thereby, the test determines a likelihood *p*-value for an observed number of cases appearing in a window of fixed width as the window is moved along the time axis studied (2003). Observed and expected cases, the latter calculated using the historical data (1998–2002), were compared with a null hypothesis that states cases occur at random, evaluated against the alternative hypothesis that states cases cluster in certain time periods. In this evaluation, a Poisson distribution was assumed because a positive MRSA finding is a rare and irregular event. Clusters of two or more infected/colonized patients or colonized staff on the same ward or wards in close contact (e.g., interdisciplinary intensive care units) occurring within a 2-wk window and harboring the same MRSA isolate according to the *spa* typing, were identified as significant at the 5% level. These statistically confirmed clusters were then used as the “gold standard” for comparing the various alert mechanisms. Non-significant clusters were considered to be sporadic occurrences.

### Early Warning Algorithms

Every MRSA isolate obtained in 2003 was examined in a prospective analysis by applying descriptive epidemiologic parameters such as time, place, and person. When two or more MRSA isolates were detected within a 2-wk window on the same ward or on wards having close contact, the resulting alert was regarded as a “frequency alert” and allocated to a “frequency cluster.” If MRSA isolates also shared an identical *spa* type, the allocation to “clonal alerts” and associated “clonal clusters” was triggered. An ICP, which is a panel consisting of two physicians and four infection control nurses who meet weekly and hold additional meetings when an outbreak occurs, rate the findings as “ICP alerts” and “ICP clusters,” respectively. When feasible, the area of surveillance is widened and an investigation initiated. The ICP uses microbial data and data from patients' charts to reach their decisions but are blind to the occurrence of an outbreak on the basis of *spa* typing results.

### Statistical Analysis

Sensitivity, specificity, positive and negative predictive values (PPV, NPV), and pre-test probability were determined as described by Sackett et al. [23]. The pre-test probability is defined as the proportion with the target disorder (MRSA cluster) in the population at risk (MRSA positive) at a specific time interval. Two-tailed, 95% CIs were calculated to assess sensitivity, specificity, PPV, and NPV using a normal approximation for the pertinent (binomial) distribution. The chi-square distribution, with one degree of freedom, was used to determine the significance of the differences in these parameters.

## Results

Table 1 summarizes the important epidemiological indicators for MRSA at the UHM. The overall prevalence of MRSA cases was 0.17 per 100 admissions and the relative risk of acquiring MRSA increased 4-fold during the study period. The annual number of patients with MRSA bacteremia reached a peak in 2003 with six patients. The average turn-around time for *spa* typing under routine laboratory conditions was 2.4 d. Of the 557 MRSA isolates tested, 549 (98.6%) could be typed using *spa* sequencing, and the eight strains, which could not be typed, were excluded from the analysis. A total of 79 different *spa* types were identified in samples collected for the period 1998–2003. The discriminatory power of *spa* typing was 91.8%. Table 2 shows the frequency with which the various *spa* types were isolated at UHM and nationally. *spa* types t003, t004, t001, and t032 were the types most frequently isolated during the study period and accounted for 52.9% of all cases. In Table 2 the typing results are also brought into a global epidemiological context as defined by PFGE and MLST [11,24–26]. The dynamics, expressed on an annual basis of the epidemic MRSA clones at UHM and in Germany as a whole, are depicted in Figure 1. In general, the findings for UHM followed the national trend, i.e., the number of “Barnim” and “Rhine Hesse” MRSA clones increased in parallel throughout the study period. As shown by data for the “Southern German” MRSA clone, the fluctuation at the regional level was more marked, and this is probably due to the smaller number of cases.

**Figure 1:**
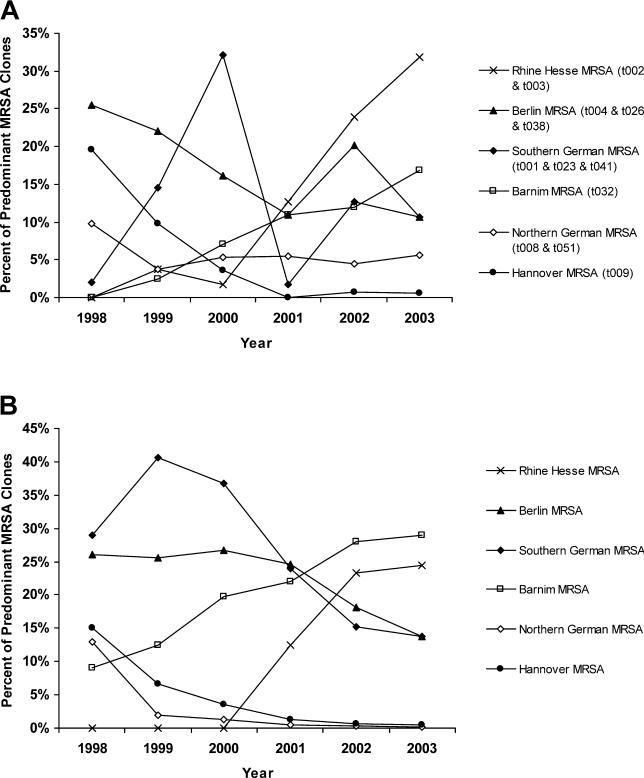
Annual Dynamics of Epidemic MRSA Clones at the UHM as Defined by *spa* Typing (A) and in Germany by PFGE (B) from 1998 to 2003

**Table 1:**
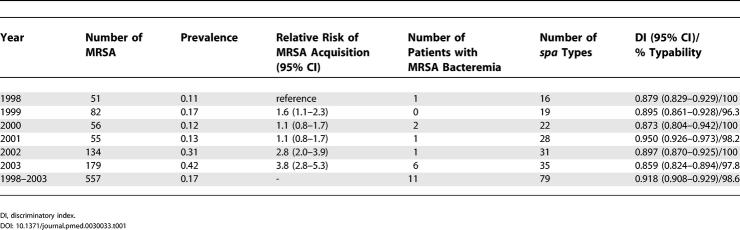
Important Epidemiological Indicators for MRSA at the UHM (1998–2003)

**Table 2:**
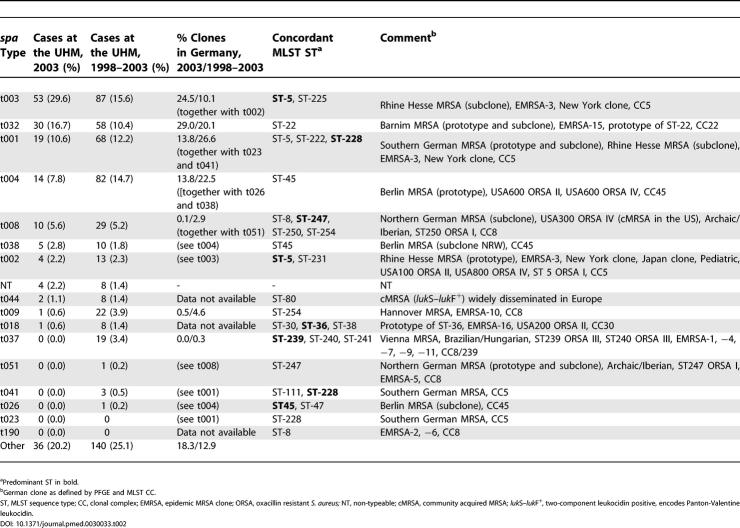
Most Frequent *spa* Types and Epidemiological Background (PFGE, MLST) at the UHM and in Germany

In 2003, a total of 175 MRSA isolates (154 patients, 21 staff) could be typed, and these comprised 34 different *spa* types. The results of the year 2003 scan test analysis are shown in Table 3. The encoded name of the clinic/ward was derived from the location of the first MRSA isolated in a particular cluster. In total, there were 42 MRSA isolates forming 13 significant clusters and representing seven different *spa* types, but five clusters involved *spa* type t003, a common *spa* type in our hospital (29.6% of all MRSA in 2003). The average time-span of the clusters was 10 d (range 1–31 d) and the number of isolates in each cluster ranged from 2–11 (mean 3.2). Six clusters were located on a single ward, whereas seven other clusters were located at sites distributed throughout a clinic.

**Table 3:**
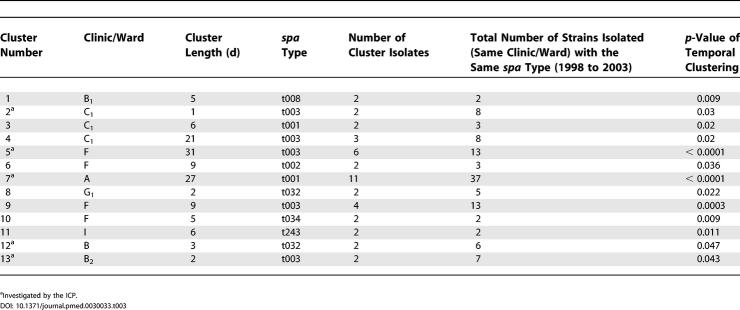
Significant MRSA Clusters in 2003 (Chronologically Ordered) Identified by Scan Test Statistics (*p* < 0.05)

In the prospective analysis of all MRSA in 2003 using the various alert procedures, there were 106 frequency alerts assignable to 31 frequency clusters (Table 4). A total of 36 clonal alerts, comprising 20 clonal clusters, were triggered by the early warning system. The ICP called 22 ICP alerts corresponding to nine ICP clusters, but in only five clusters (the two largest clusters, numbers five and seven were included) was the recognition of an existing outbreak and the need for further investigation correct. The four other clusters arose from false alerts by the ICP. In Table 4, the alerts triggered by the various methods are categorized as true or false alerts using the alerts for the 13 significant “true” clusters. The sensitivity, specificity, PPV, and NPV, and where appropriate, the 95% CIs for the various alert methods, are displayed in Table 5. Because of the high number of false-positive frequency alerts (*n* = 77), the specificity of the frequency and clonal methods (47.2% and 95.2%, respectively) differed considerably. The ICP alerts had the highest specificity, but the number of false-negative alerts (eight of 13 confirmed clusters were missed) led to the lowest sensitivity (62.1%). Given a pre-test probability of 24%, the PPV (same as the post-test probability) of the ICP and clonal alerts was above 80%, whereas the PPV of the frequency alert was only 27.4%. There were no significant differences in specificity and PPV between clonal and ICP alerts. Frequency alerts were significantly less sensitive (*p* < 0.001) and less accurate in making positive predictions than clonal and ICP alerts.

**Table 4:**
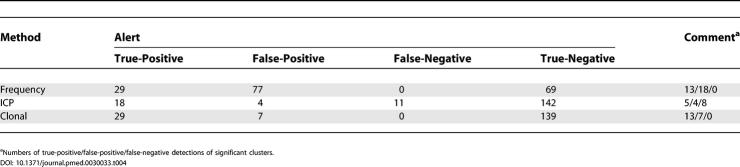
True and False Alerts/Clusters with the Various Methods Determined Using the 29 Alerts Associated with the 13 Significant Clusters in 2003

**Table 5:**
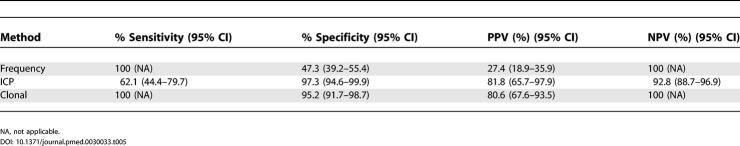
Sensitivity, Specificity, PPV, and NPV of the Various Alert Methods (24% Pre-Test Probability) in 2003

## Discussion

In this paper we have presented a new method for prospective MRSA outbreak surveillance in a hospital that uses case and molecular typing data. Historically, MRSA outbreak detection in hospitals has relied on the watchful eyes of physicians and other health-care workers. However, the increasing availability of timely electronic surveillance and molecular typing data raises the possibility of earlier outbreak detection and intervention if suitable analytic methods are found.

Germany belongs to a group of Western European countries with an intermediate level of MRSA (approximately 20% of all *S. aureus* diagnosed in laboratories are MRSA positive). However, the isolation rate has increased significantly in recent years [27]. Although the MRSA laboratory isolation rate in UHM of 6.4% in 2003 is still rather low in comparison with other German hospitals, the relative risk of acquiring MRSA within this hospital facility rose significantly during the study period (Table 1). Furthermore, the absolute risk will also probably rise because of epidemiological pressure and the rising prevalence of MRSA in Germany as a whole (Table 2 and Figure 1). It is clear that control of MRSA is a pressing concern where new concepts are needed, and therefore we studied *spa* typing in combination with an automatic early warning algorithm to detect MRSA clusters at UHM.

We showed that the feasibility and speed with which it was possible to carry out *spa* typing was highly satisfactory. The discriminatory power, however, was lower than previously reported, probably because only a local strain collection was analyzed [12,13]. Although not examined by us, the high intra- and inter-laboratory reproducibility of 100% and the robustness of the method have recently been documented (Aires-de-Sousa et al., unpublished data). Moreover, there is a high concordance of results between *spa* and PFGE, microarray and MLST [11,12]. The practicability of using *spa* in short-term epidemiological studies has been questioned because differences in PCR amplicon sizes in related strains was thought to imply instability in the target gene [28]. In the meantime, however, there has been a plethora of publications demonstrating the value of *spa* in the investigation of MRSA outbreaks, e.g., [10,14]. Moreover, it has recently been shown that *spa* data not only contain information on short-term, but also long-term evolutionary events, as observed in whole repeat duplications and deletions [12,29]. Because of the steady fall in the cost of DNA sequencing and an average hands-on time of only 20 min per sample (determination of both strands of DNA and processing ten samples in parallel), this technique is within the capability of even small laboratories [30].

The present study has compared three early warning algorithms for the detection of nosocomial MRSA outbreaks before limited clusters of preventable MRSA transmissions develop into larger outbreaks. The evaluation of an early warning system, however, is difficult because there is no accepted “gold standard” and it is likely that no system will be completely reliable [31]. Therefore, we chose to combine epidemiological and molecular typing data with statistical analysis to provide an objective measure of performance between the varied approaches. In this approach, by definition, the sensitivity and NPV of the frequency and clonal alert methods will always be 100%. This means that the ICP method will give results that are less sensitive, or at best, of only equal sensitivity to those of the automated methods. Infection and colonization with MRSA were given equal status since both can lead to further transmissions. The typing data accumulated since 1998 enables the significance of *spa*-type clustering with respect to time to be calculated for all those occasions when there is a suspicion of an outbreak. By excluding all non-significant clusters, it was possible to reduce the likelihood that two or more MRSA with the same *spa* type, coincidentally isolated on the same or related wards within the 2-wk window, would be counted as correct. A 2-wk window is approximately 1.5 times as long as the mean duration of hospitalization in the UHM hospital. A 4-wk time window yielded similar results (unpublished data).

An outbreak can be defined as (i) two or more cases of infection by a common agent that are linked epidemiologically. However, this definition has usually limited practical relevance in the identification of outbreaks, because it presupposes that detailed epidemiological and typing data are available as soon as the outbreak occurs. Thus, in more practical terms, an outbreak is often defined operationally as (ii) an increase in the number of cases above expected levels [32]. Historical data can be used to calculate a baseline and an alert is given when the number of cases exceeds a certain threshold. Early warning systems at national levels are based on this definition of an outbreak and have already implemented this approach successfully [33,34]. A similar approach has also been used in hospitals, e.g., using 2-fold standard deviation and monthly increase algorithms for detecting clusters of nosocomial infections [31]. In using the 2-fold standard deviation algorithm, the threshold for a suspected outbreak is defined as the mean of all previous cases per unit time plus two standard deviations. The monthly increase algorithm triggers an alert if there is either a 100% increase in the number of observed cases in the current month compared to the monthly totals for the two previous months, or a 50% increase over a three-month period. In the case of MRSA, however, infections/colonizations occur infrequently and irregularly. Applying the 2-fold standard deviation and monthly increase algorithms to our data (including typing data) resulted in delayed alerts for cluster detection indicating that they were insufficiently sensitive (unpublished data). In order to improve the detection of MRSA clusters and to avoid delay, we applied the first of the two definitions of an outbreak mentioned above, i.e., two or more cases of infection/colonization that are linked epidemiologically, because discriminatory typing details of cases were rapidly available.

The ability of the current procedures in hospitals to prevent nosocomial infections and to recognize nosocomial outbreaks often depends on the manual review of laboratory results and surveillance by the ICP. However, this review process is resource-intensive because duplicate isolates must be eliminated, results must be correlated with patients' charts, patient locations within the hospital must be tracked, and related events must be correlated and monitored [6]. Not surprising, many minor transmissions of MRSA infections amenable to intervention go undiscovered. In this study, only five of the 13 “true” clusters were detected as clusters by visual screening of laboratory reports by the ICP (Table 3 and 4). However, the ICP alerts had the highest PPV (81.8%) because of their high specificity. On the other hand, frequency alerts with a sensitivity of 100% detected every cluster. However, the high number of false-positive alerts giving the lowest PPV (27.4%) clearly demonstrates that this method is unsuitable (also shown by the significant differences in specificity and PPV compared with clonal and ICP alerts). Clonal alerts combine the best of both methods, i.e., high specificity and high sensitivity with a PPV comparable to that of ICP (no significant difference). In comparison to ICP, only a few more false-positive alerts were triggered, and more clusters, especially the smaller ones, were detected (Table 5). The data also indicated that surveillance conducted in the laboratory has the advantage in that clusters occurring throughout the hospital can be identified at a single, central data collection point. Further advantages are its speed (<3 d after detection of MRSA) and the portable nature of data generated by *spa* typing permitting the differentiation between outbreaks and pseudo-outbreaks and the central coordination of a suitable response in real-time [14]. Cost-benefit analyses have demonstrated that the cost of MRSA infections far exceeds those costs involved in active surveillance and isolation procedures in a hospital [35,36]. Whether the expenditure required for *spa* typing is less than that for the labor-intensive manual review of patients' charts and laboratory results needs to be determined.

Since the second half of 2004, when the study was finished and data analyzed, the Ridom StaphType software v. 1.5 beta that features an automated early warning based on clonal alerts came into routine use in our laboratory. A data-driven “re-admission alert” triggered by a hospital information system, which identifies the re-admission of any patient previously colonized or infected with MRSA, could enhance the accuracy of such a system [37].

All methods described above are based on underlying rules or seek predefined patterns. The advantages of these hypothesis-based methods are the high sensitivity and specificity achievable. However, a rapid method with a high discrimination involving gene typing is necessary to attain such a high specificity. Due to the predefined rules, unusual patterns of outbreaks might go undetected (e.g., retarded epidemics). A different approach is employed by data mining, i.e., knowledge discovery in databases [6]. Data-mining uses techniques based on computer science and statistics to search large event spaces (data warehouses) for interesting patterns that would otherwise have gone undetected by traditional analysis. These “discovery” models are independent of an underlying hypothesis, but are usually less sensitive and specific [38].

There are a number of limitations within the proposed method. There is no definitive proof available, with any method, to authenticate a MRSA transmission event. Furthermore, the “gold standard” used incorporates elements of the diagnostic test under study. Finally, if the epidemiological pressure of a certain clone changes rapidly, temporal clustering could fail and false-positive (e.g., as possible in the case of the “Rhine Hesse” MRSA clone) or false-negative clusters could be recorded.

Examining the occurrence of MRSA clusters in the way described here not only provides a useful early warning system, but can also be used to model infection dynamics and estimate important epidemiological parameters, e.g., cross-transmission rates [39,40]. Time series and typing data was used by Grundmann et al. with scan test statistics and risk factor analysis to show that the incidence of infection can be related to staffing levels [41]. In addition, molecular typing data can also be incorporated into geographical information systems combined with space-time scan statistics analysis on a regional and national level [42,43].

In conclusion, a surveillance method based on *spa* typing and automated alerts is useful as an early warning system in a hospital and is at least comparable to classical epidemiological approaches. We have shown that the combined use of medical informatics and molecular laboratory techniques makes intervention possible before limited clusters of preventable MRSA transmissions expand into outbreaks.

**Patient Summary**

**Background**

Everyone carries many types of bacteria on or in their bodies; *Staphylococcus aureus* is a normal bacteria for people to carry. About 25% to 30% of people have it, usually in the nose. It is usually harmless; however, this bacterium can also cause infections—especially in people who are otherwise unwell, or who have surgery. These infections need to be treated with antibiotics. Methicillin-resistant *S. aureus* (MRSA) is an increasing problem in much of the developed world because, unlike other types of these bacteria, MRSA cannot be killed by most of the usual antibiotics that are used, such as methicillin. Without treatment, staphylococcal infection can become very severe.

**Why Was This Study Done?**

MRSA is a particular problem in hospitals, where there is a need to be able to identify infected and colonized people quickly and isolate and treat them. These researchers wanted to test for the best way of identifying early clusters of MRSA outbreaks, which are more serious than just single cases and are an indication of hygiene deficiencies.

**What Did the Researchers Do and Find?**

Between 1998 and 2003 the researchers analysed 557 MRSA strains from staff and patients admitted to one German university hospital. They collected information about the characteristics (in space and time) of these people, and genetically identified each of the strains. They then looked for the most efficient way to identify an outbreak, including assessment of the risk by specially trained hospital staff, with and without genetic analysis. They also assessed a specially designed computer programme (developed by some of the authors), which combined the genetic type of the MRSA as well as details about the outbreak, such as the characteristics of the patients infected. They found that the most efficient and reliable method to identify outbreaks was to combine the genetic type of the MRSA with details about the outbreak, using the computer programme tested.

**What Do These Findings Mean?**

The computer programme seems to be more efficient than other methods tested here in identifying when an outbreak is likely to occur. However, this is the first test of this method, and before being adopted more widely, further testing is needed in different settings and by other researchers.

**Where Can I Get More Information Online?**

Medline Plus has many links to pages of information on different staphylococcal infections:

http://www.nlm.nih.gov/medlineplus/staphylococcalinfections.html

The Centers for Disease Control in the United States has a patient information sheet on MRSA:

http://www.cdc.gov/ncidod/hip/aresist/ca_mrsa_public.htm

The Health Protection Agency in the United Kingdom has a leaflet on MRSA aimed at patients:

http://www.hpa.org.uk/infections/topics_az/staphylo/mrsa_leaflet.htm

## Abbreviations

CI: confidence interval
ICP: infection control professional
MLST: multi-locus sequence typing
MRSA: methicillin-resistant *Staphylococcus aureus*
NPV: negative predictive value
PFGE: pulsed-field gel electrophoresis
PPV: positive predictive value
*spa*: *S. aureus* protein A gene
UHM: University Hospital Münster
